# Velocity unwrap for high resolution slice-selective Fourier Velocity Encoding using spiral SENSE

**DOI:** 10.1186/1532-429X-14-S1-W25

**Published:** 2012-02-01

**Authors:** Jennifer A Steeden, David Atkinson, Alexander Jones, Vivek Muthurangu

**Affiliations:** 1 Institude of Cardiovascular Science, UCL, London, UK; 2 Centre for Medical Imaging, UCL, London, UK

## Background

In patients with stenoses, it is desirable to accurately measure peak velocity (Vmax). Unfortunately, phase-contrast MR (PCMR) tends to underestimate peak velocities. Fourier Velocity Encoding (FVE) can measure peak velocities in MRI, but is not commonly used due to long acquisition times.

We have developed a FVE sequence that combines spiral trajectories with parallel imaging (SENSE), partial-Fourier acquisition and a novel velocity-unwrap technique. The aim of this study is to validate this sequence.

## Methods

FVE was performed using a uniform-density spiral trajectory with 16 interleaves (table 1). Parallel imaging was applied (R=4) and reconstructed using an iterative SENSE algorithm. Partial-Fourier was performed in kv (67%) with a homodyne reconstruction.

**Table 1:** Institude of Cardiovascular Science

	lr-PCMR	hr-PCMR	FVE21	FVE41
TE/TR (ms)	~2.2/5.0	~2.2/5.0	~2.5/9.3	~3.5/10.3

Readouts	Cartesian	Cartesian	Spiral: 16 interleaves	Spiral: 16 interleaves

Matrix Size	128	256	192	192

Image FOV (mm)	320	320	450	450

kv positions acquired	-	-	14	15

Reconstructed velocity levels	-	-	21	41

Total Scan Duration (heartbeats)	15	108	15	15

Spatial resolution (mm)	~2.5	~1.3	~2.3	~2.3

Temporal resolution (ms)	~40	~30	~37	~41

Velocity resolution (cm/s)	-	-	30-75	18-38

Velocity-unwrap: By acquiring the centre half of kv-positions, reconstructed data is aliased in v-space. Acquiring one additional kv-position with the full VENC, and reconstructing this using traditional PCMR provides information about the direction of flow (on a pixel-by-pixel, frame-by-frame basis). This allows accurate unfolding of velocity data.

In-vitro: A pulsatile flow pump was connected to a tube phantom (diameter 13mm) with a stenosis of 6mm. At 15 different flow rates, Vmax was measured using; 1) ultrasound (US), 2) low-resolution PCMR (lr-PCMR), 3) high-resolution PCMR (hr-PCMR), 4) FVE with SENSE and partial-Fourier with 21 reconstructed velocities (FVE21) and 5) FVE with SENSE and partial-Fourier, plus velocity-unwrap giving 41 reconstructed velocities (FVE41). SNR estimates were compared between FVE21 and FVE41.

In-vivo: Six patients with stenoses were also assessed (3M:3F; 31±21years).

## Results

In-vitro: There were no statistically significant differences between Vmax measured using US and FVE (table 2). However both PCMR sequences showed statistically significant underestimation of Vmax compared to US. This is particularly true of lr-PCMR, which underestimated Vmax by >0.5m/s.

**Table 2:** 

	Echo	lr-PCMR	hr-PCMR	FVE21	FVE41
In-vitro					

Peak velocity (cm/s)	441±144	375±133^	398±136^	447±140	443±144

Bias* (cm/s)	-	-66	-42	+7	+3

Limits of agreement* (cm/s)	-	-26 to -105	-10 to -75	+28 to -14	+17 to -12

Correlation coefficient* (r)	-	0.9926	0.9949	0.9977	0.9987

Estimated SNR	-	-	-	5.2±2.6	4.4±3.9

In-vivo					

Peak Velocity (cm/s)	255±71	228±42	234±644	266±67	257±62

Bias* (cm/s)	-	-27	-21	+10	+1

Limits of agreement* (cm/s)	-	+67 to -121	+63 to -106	+74 to -53	+47 to -46

Correlation coefficient* (r)	-	0.7717	0.8269	0.8957	0.9463

* Calculated with echo ^Value is significantly different (ANOVA) from echo (P<0.05)

In-vivo: As in-vitro, PCMR underestimated Vmax. There were no statistical differences between Vmax measured using US and FVE sequences. However there was a trend towards FVE21 overestimating Vmax.

## Conclusions

FVE allows more accurate assessment of Vmax than PCMR as it measures a velocity spectrum per pixel, rather than the average velocity. We have demonstrated that it is possible to achieve high resolution FVE within a short breath-hold by combining spiral trajectories, parallel imaging, partial-Fourier and velocity-unwrap. This sequence was shown to be significantly more accurate than PCMR in-vitro and in-vivo. Furthermore using the novel velocity-unwrap technique there was a trend towards higher accuracy due to higher velocity resolution. Thus, the sequence may be able to replace US in assessment of Vmax.

## Funding

JAS: EPSRC PhD+.

VM: BHF.

